# Phylodynamics beyond neutrality: the impact of incomplete purifying selection on viral phylogenies and inference

**DOI:** 10.1098/rstb.2023.0314

**Published:** 2025-02-20

**Authors:** Katia Koelle, David A. Rasmussen

**Affiliations:** ^1^ Department of Biology, Emory University, Atlanta, GA 30322, USA; ^2^ Emory Center of Excellence for Influenza Research and Response (CEIRR), Atlanta, GA 30322, USA; ^3^ Department of Entomology and Plant Pathology, North Carolina State University, Raleigh, NC 27607, USA; ^4^ Bioinformatics Research Center, North Carolina State University, Raleigh, NC 27607, USA

**Keywords:** viral phylodynamics, phylodynamic inference, incomplete purifying selection, deleterious mutations

## Abstract

Viral phylodynamics focuses on using sequence data to make inferences about the population dynamics of viral diseases. These inferences commonly include estimation of growth rates, reproduction numbers and times of most recent common ancestor. With few exceptions, existing phylodynamic inference approaches assume that all observed and ancestral viral genetic variation is fitness-neutral. This assumption is commonly violated, with a large body of analyses indicating that fitness varies substantially among genotypes circulating in viral populations. Here, we focus on fitness variation arising from deleterious mutations, asking whether incomplete purifying selection of deleterious mutations has the potential to bias phylodynamic inference. We use simulations of an exponentially growing population to explore how incomplete purifying selection distorts tree shape and shifts the distribution of mutations over trees. We find that incomplete purifying selection strongly shapes the distribution of mutations while only weakly impacting tree shape. Despite incomplete purifying selection shifting the distribution of deleterious mutations, we find little discernible bias in estimates of viral growth rates and times of the most recent common ancestor. Our results reassuringly indicate that existing phylodynamic inference approaches that assume neutrality may nevertheless yield accurate epidemiological estimates in the face of incomplete purifying selection. More work is needed to assess the robustness of these findings to alternative epidemiological parametrizations.

This article is part of the theme issue ‘'"A mathematical theory of evolution": phylogenetic models dating back 100 years’.

## 1. Introduction

This article contributes to a special issue commemorating G. Udny Yule’s contribution to the mathematical phylogenetics literature [1]. Published 100 years ago in *Philosophical Transactions B*, Yule’s paper ‘A Mathematical Theory of Evolution’ presents a simple generative model for evolutionary diversification. Yule interfaced this model with taxonomic data to estimate model parameters such as speciation rates. Although the details differ considerably, the overall structure of Yule’s approach is reflected in viral phylodynamic analyses today. These analyses commonly involve the specification of a generative model and estimation of model parameters such as growth rates and reproduction numbers [2–4]. Model parameters are estimated using reconstructed time-resolved viral phylogenies, which nowadays are generally jointly inferred together with the model parameters from sequence data [5–7].

Phylodynamic analyses have also relied on many of the same modelling assumptions as those adopted by Yule in his classic 1925 paper. Most notably, Yule’s model assumes that species-creating mutations are either ‘viable’ or ‘non-viable’ and that all species are equally likely to give rise to the next species. These assumptions lead to the condition of exchangeability being satisfied between species. Similarly, most phylodynamic models based on standard coalescent and birth–death processes ensure exchangeability by assuming all sequence variation is neutral, such that all viral lineages share the same fitness regardless of their genotype [8–10]. While certain models like the multi-type birth–death model allow fitness to vary between lineages based on their genotype, these non-neutral models come at a considerable computational cost even when the number of modelled types is small [11–14]. As a result, the most widely used methods for reconstructing epidemic dynamics from viral sequence data, including those that yield Bayesian Skyline [10] and Skygrid [15] plots, all assume pathogen sequences evolve neutrally.

The assumption that viral mutations are fitness-neutral is in direct conflict with a robust set of empirical data. Here, we first briefly review existing analyses that indicate that sublethal deleterious mutations commonly circulate and often contribute substantially to fitness variation between co-circulating viral lineages (§2). By drawing from the theoretical population genetic literature, we then summarize the impact that incomplete purifying selection is known to have on phylogenetic tree shape, as well as on the distribution of mutations on these trees (§3). We then examine the impact that tree shape distortion alone (§4) and with non-neutral mutation distributions (§5) has on viral phylodynamic inference when purifying selection is incomplete. In §6, we assess the sensitivity of our results to sequence sampling times. Finally, we discuss our results in §7.

## 2. Empirical evidence for incomplete purifying selection in viral populations

Across the diversity of life, most mutations are known to be deleterious. While some of these mutations are lethal deleterious (‘non-viable’), many are sublethal deleterious [16]. In RNA viruses, this pattern also holds true: the overwhelming majority of mutations exact a fitness cost [17–19], with approximately 30% being lethal deleterious and the remaining 70% of mutations being sublethal deleterious. While purifying selection tends to remove deleterious mutations from viral populations over time, selection is generally not strong enough to immediately purge all deleterious mutations. This is especially true if the fitness effects of deleterious mutations and infected population sizes are both small, in which case purifying selection may be weaker than genetic drift. Furthermore, because viral mutation rates are generally high, the rate at which deleterious mutations enter a viral population can exceed the rate at which purifying selection purges them. Many deleterious mutations can therefore co-circulate in viral populations, and these mutations can cause viral fitness to vary substantially between lineages.

The imprint of incomplete purifying selection acting on deleterious mutations can be observed in reconstructed viral phylogenies. For example, a phylogenetic analysis of 143 RNA viruses showed that the ratio of nonsynonymous to synonymous substitution rates on external lineages (i.e. branches leading to sampled tips) was larger than that of internal lineages [20]. A simple explanation for this pattern is that purifying selection has had time to filter out lineages carrying deleterious mutations deeper in the tree but not among the more recently sampled lineages. Incomplete purifying selection may also explain why molecular clock rates estimated from viral sequence data are often found to be time-dependent, with higher rates estimated from samples collected over shorter timescales such as when samples are collected only in the recent past [21–24]. Consistent with this explanation Ghafari *et al*. [25] estimated that clock rates are 2–4 times higher along external branches relative to internal branches in pandemic H1N1 influenza and SARS-CoV-2 phylogenies. Other studies allowing for branch-specific molecular clocks did not infer such extreme differences in clock rates, with clock rates varying more within internal/external branches than between these groups of branches [26]. Such extreme differences in clock rates between external and internal branches may therefore only arise when viral sequences are sampled over a relatively short timespan, such as early on during an emerging epidemic [25,27].

Several additional studies provide more indirect evidence for incomplete purifying selection in RNA virus populations. One such study focused on predicting clade frequencies of influenza A virus from one season to the next [28]. To predict clade frequency changes, relative fitness values of strains were estimated. For accurate prediction, the authors needed to incorporate fitness costs of amino acid changes that occurred outside of epitope regions (and were thus unlikely to impact antigenicity). Finally, a previous study of ours [29] found that incomplete purifying selection could explain influenza A H3N2’s slender phylogeny and its punctuated antigenic evolution [30]. Among competing antigenic variants, transiently circulating deleterious mutations create additional variation in background fitness in which new antigenic or other beneficial mutations arise, such that beneficial mutations need to occur in higher fitness backgrounds (‘jackpot events’) carrying fewer deleterious mutations in order to displace competing variants. In the absence of circulating deleterious mutations, our simulations instead predicted unrealistic levels of viral antigenic diversification in the long term.

## 3. Brief review of the impact of incomplete purifying selection on phylogenies

**Figure 1.**
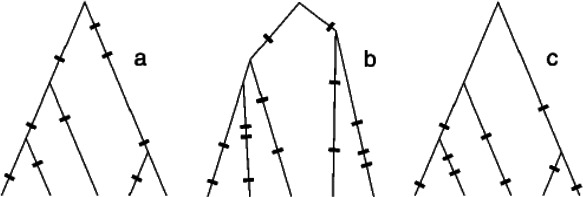
Distinct ways in which purifying selection can impact genealogies, reproduced with permission from [31]. (*a*) A neutral genealogy in a constant sized population. Both the shape of the tree and the distribution of mutations on the tree are consistent with the neutral expectation. (*b*) A genealogy that differs only in tree shape from the one expected under neutrality. The distribution of mutations is consistent with the neutral expectation. (*c*) A genealogy that differs only in the distribution of mutations from one expected under neutrality. The tree shape is consistent with the neutral expectation.

Incomplete purifying selection can impact genealogies in two different ways [31] (figure 1). It can distort the shape of a genealogy away from its neutral expectation (figure 1*b* versus figure 1*a*), where shape is defined broadly as a tree’s distribution of internal and external branch lengths as well as its symmetry in terms of the number of descendants each ancestral lineage gives rise to. It can also impact the distribution of mutations across a genealogy (figure 1*c* versus figure 1*a*). Early studies that considered one-locus, two-allele models found that purifying selection had only a weak, if at all discernible, impact on tree shape [32,33]. Later studies transitioned to using simulation-based models that allowed for a larger number of sites and fewer assumptions relating to the fitness costs of deleterious mutations [31,34,35]. These studies corroborated earlier findings that, in certain regions of parameter space, incomplete purifying selection may only slightly impact the shape of genealogies. For example, Williamson and Orive [31] calculated metrics such as the proportion of tree length composed of external branches from simulated genealogies over a range of deleterious mutation fitness costs and found that this metric was not substantially impacted by incomplete purifying selection, regardless of the assumed fitness cost of mutations. Overall tree lengths were also not substantially impacted when mutations exacted either a small or a very large fitness cost. However, when mutations exacted an intermediate fitness cost, overall tree length was smaller than under neutrality. Similarly, Maia *et al*. [34] found that genealogies derived from contemporaneously sampled individuals exhibited little asymmetry when mutations carried either a small or a large fitness cost. When mutations exacted an intermediate fitness cost, however, genealogies would become more asymmetrical, an effect that became even more pronounced at large sample sizes. Consistent with these results, Seger *et al*. [35] found that the shapes of genealogies were maximally distorted when fitness costs of mutations were of intermediate size.

Incomplete purifying selection can further shift the temporal distribution of coalescent (branching) events. Many theoretical models suggest that purifying selection will act to reduce historical effective population sizes, thereby increasing the rate at which lineages coalesce [35–38]. Intuitively, this reduction in effective population size arises because individuals sampled at present have a higher probability of descending from more fit ancestors [37,39]. Thus, the ‘effective pool’ of ancestors from which sampled individuals can descend is reduced relative to neutral expectations where all historical individuals have equal probability of producing sampled descendants. While it has been suggested that the effects of purifying selection on coalescent times can be accounted for by simply rescaling historical effective population sizes [40], others have argued that no such simple rescaling exists [37]. In either case, neutral phylodynamic models that infer time-varying effective population sizes (e.g. Bayesian Skyline models) or that explicitly infer growth rates [10,40,41] may be prone to biases in demographic inference owing to shifts in the temporal distribution of coalescent events caused by purifying selection.

In addition to any effects that purifying selection may have on tree shape, purifying selection has the potential to dramatically shift the distribution of mutations on a genealogy. Specifically, the proportion of mutations that fall on external branches relative to internal branches can be substantially higher in the presence of purifying selection than under selective neutrality [31], with this proportion increasing at higher strengths of selection. The clustering of mutations towards the tips of genealogies violates the assumption under neutrality that mutations accumulate at a uniform rate across a tree such that the number of mutations along each branch is Poisson-distributed. This can bias branch lengths estimated under the assumption of a constant molecular clock, which in principle could skew coalescent estimates of parameters such as migration rates, effective population sizes and population growth rates [31].

## 4. Impact of tree shape distortion on phylodynamic inference

**Figure 2.**
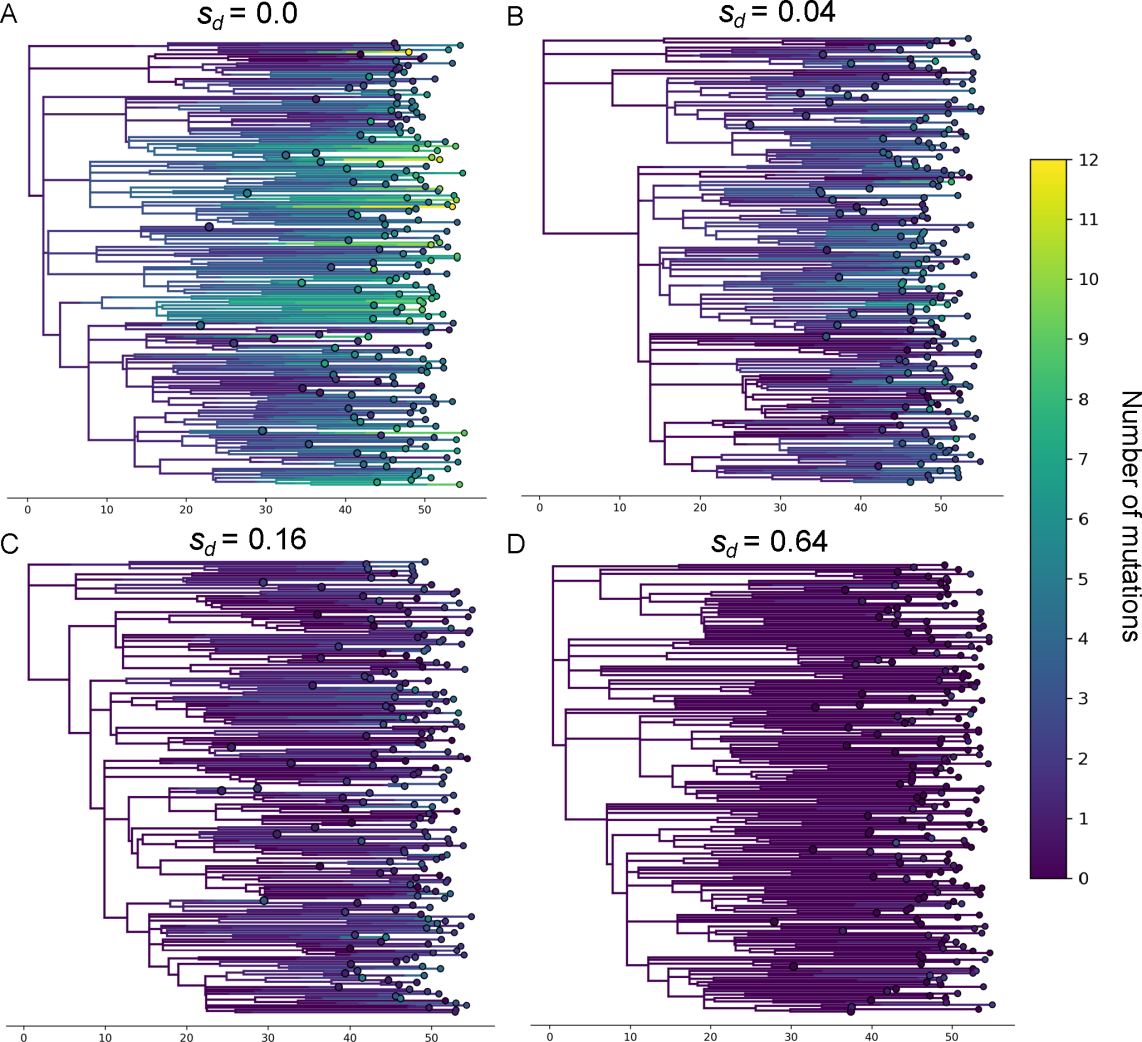
Viral genealogies simulated under different mutational fitness costs $s_{d}$. Branches are coloured according to the number of mutations they carry. Note that the distribution of mutations over the tree can also be observed through these changes in colour.

Based on the review above, we first hypothesized that viral phylogenies inferred during an emerging epidemic have the potential to be distorted in shape, and thus, that phylodynamic inference may yield inaccurate epidemiological estimates even when only considering the impact that incomplete purifying selection has on tree shape, without considering additional biases owing to distortions in the distribution of mutations. To test this hypothesis, we first simulated exponentially growing viral populations, each for T=55 days across a range of assumed mutational fitness costs ($s_{d}$; see §8). Epidemiological and evolutionary parameters were chosen to reflect a directly transmitted respiratory pathogen such as SARS-CoV-2. From each simulation, we obtained the true viral genealogy of a random sample of 200 infected hosts (sampled at recovery). Representative viral genealogies simulated under different mutational fitness costs are shown in figure 2.

**Figure 3.**
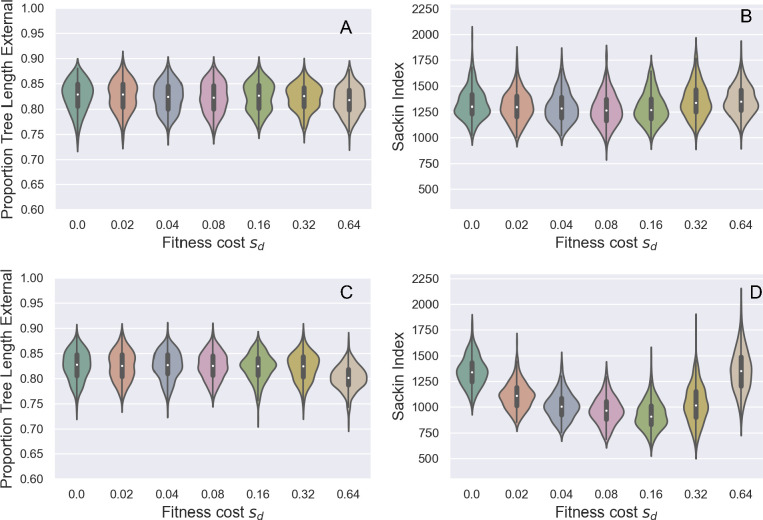
The impact of purifying selection on tree shape across a range of assumed deleterious fitness costs ($s_{d}$). (*a*) Proportion of total tree length that falls on external branches. (*b*) Sackin index of imbalance. In (*a*) and (*b*), the mutation rate was set to m=0.30 mutations per genome per transmission. (*c*) Proportion of tree length that falls on external branches. (*d*) Sackin index of imbalance. In (*c*) and (*d*), the mutation rate was set to m=3.00 mutations per genome per transmission. Violin plots show the distribution of the tree statistics for 100 ‘successful’ simulations of the forward model, as defined in §2.

From these genealogies and our other simulated genealogies, we calculated two statistics: the proportion of the total tree length that falls on external branches and the Sackin index of imbalance. For the neutral forward simulations ($s_{d}=0$), the proportion of the total tree length that falls on external branches was approximately 82.5% (figure 3*a*). This proportion is substantially higher than the neutral expectation under a constant-sized population sampled at a single time point, where the expectation is approximately 24% [42]. The higher value of this statistic in our simulations is due to exponential growth, which acts to make a phylogeny more ‘star-like’ [43,44], with shortened internal branches and lengthened external branches relative to a genealogy from a constant-sized infected host population. An additional reason for this higher value may be because samples in our simulation were taken across time, rather than at a single timepoint. Interestingly, the proportion of the total tree length that falls on external branches does not change appreciably even when mutations exact a high fitness cost (figure 3*a*). This result is consistent with the findings of [31], which indicated, using this same statistic, that the strength of selection does not appreciably alter branch length distributions in a constant-sized population. We further find no appreciable effect of deleterious mutations on tree (im)balance, with the Sackin index staying at a similar value across the range of mutational fitness costs considered (figure 3*b*). This is perhaps not surprising given previous work that has shown only weak to moderate impact of purifying selection on the average height of a leaf [34].

**Figure 4.**
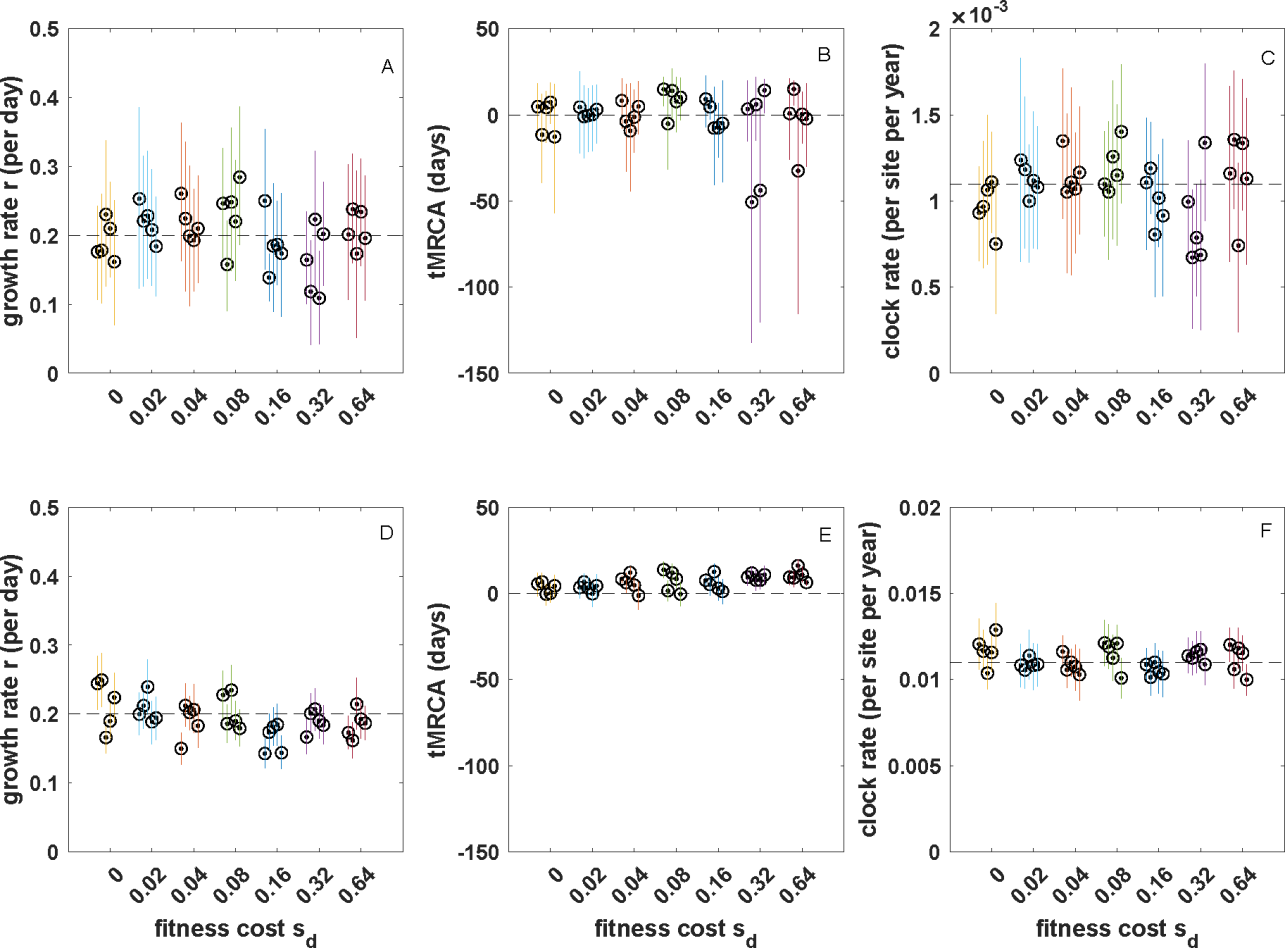
The impact of altered tree shape on phylodynamic inference across a range of mutational fitness costs. (*a*) Estimated exponential growth rates. (*b*) Estimated times of the most recent common ancestor. (*c*) Estimated clock rates. In (*a*)–(*c*), mutations were Poisson-distributed across branches under the assumption of a mutation rate of m=0.3 mutations per genome per transmission event (see §2). (*d*) Estimated exponential growth rates. (*e*) Estimated times of the MRCA. (*f*) Estimated clock rates. In (*d*)–(*f*), mutations were Poisson-distributed across branches under the assumption of a mutation rate of m=3.0 mutations per genome per transmission event. At both mutation rates, inferences from five replicate simulations are shown for each fitness cost. Circles show mean estimates. Lines show 95% highest posterior density (HPD) intervals. Dashed lines in panels (*a*,*c*,*d,f*) show true values. In panels (*b*) and (*e*), dashed lines show simulation start times of T=0 days. True tMRCA times may fall slightly later than T=0 days. MCMC (Markov chain Monte Carlo) chain length for all runs was $10^{8}$, with all effective sample sizes (ESS) exceeding 200 in all runs. Uninformative priors were used for all parameters. For ease of comparison across results, panels (*a*) and (*d*) have the same *y*-axis range and panels (*b*) and (*e*) have the same *y*-axis range.

Based on these results, we conclude that tree shape (as measured by these two tree shape statistics) is negligibly impacted by incomplete purifying selection during the early epidemic phase of an acutely infecting viral pathogen with epidemiological parameters similar to those of SARS-CoV-2. As such, we expect there to be little impact of incomplete purifying selection on phylodynamic inference when only the impact on tree shape is considered. To test this prediction, we simulated five 200-tip genealogies under each fitness cost parametrization. To generate mock sequence data, we simulated sequence evolution along these trees by randomly scattering mutations over the tree under a Poisson process as would be expected under neutrality (see §2). We then applied the coalescent exponential model [45] implemented in BEAST v. 1.10.4 [46] to these simulated sequences. From each sequence alignment, we jointly estimated the genealogy, the exponential growth rate r, the time of the most recent common ancestor (tMRCA) and the clock rate (figure 4*a*–c). Consistent with our expectations, the ‘true’ growth rate of r=0.2 per day was recovered when mutations were neutral ($s_{d}=0$). We were also able to recover the ‘true’tMRCA and the ‘true’ clock rate under the neutral model. As expected based on our tree shape findings, when mutations exacted a fitness cost, estimates of the growth rate, tMRCA and clock rate were still accurate. These results indicate that any impact that incomplete purifying selection had on tree shape did not appear to bias phylodynamic inference.

There are several reasons for why tree shape might only be negligibly impacted by purifying selection in our forward simulations. One reason is that deleterious mutations potentially never strongly impact tree shape. Another reason might be that our assumed mutation rate may be too low to allow a significant number of deleterious mutations to accumulate over the timespan of our simulations. To evaluate this possibility, we re-simulated the model with an order-of-magnitude higher mutation rate. At this higher mutation rate, the proportion of total tree length composed of external branches again does not appear to change as the fitness cost of deleterious mutation increases (figure 3*c*). However, at this higher mutation rate, the Sackin index of imbalance now appears to depend on the fitness cost of mutations (figure 3*d*). Specifically, the Sackin index first decreases with increasing $s_{d}$ (to approximately 70% of its original value), indicating that trees become more balanced, but then increases again at much higher $s_{d}$ indicating that trees become less balanced. This result is rather counter-intuitive and inconsistent with [34], since we would expect trees to be the most imbalanced at intermediate fitness costs where the most fitness variation would accumulate. We speculate that in an exponentially growing population, strong purifying selection may actually lead to more balanced trees owing to early branching lineages, with high variance in their number of sampled descendants being ‘pruned’ or replaced by more fit lineages.

As before, we scattered mutations onto five randomly selected genealogies for each of the fitness cost simulations according to their branch lengths, now using the higher mutation rate. We then generated 200 sequences for each of these genealogies and applied the coalescent exponential model to these simulated sequences. We were again able to recover the true growth rate of r=0.2 per day under all mutational fitness costs considered (figure 4*d*), as well as the tMRCAs (figure 4*e*) and the clock rate (figure 4*f*). As expected, the credible intervals were tighter for the simulations that used a higher mutation rate (figure 4*d*–*f* versus figure 4*a*–*c*) owing to less phylogenetic uncertainty under the higher mutation rate. Reassuringly, the impact of incomplete purifying selection on tree balance that is observed at intermediate levels of $s_{d}$ at the higher mutation rate (figure 3*d*) does not appear to bias growth rate estimates, tMRCA estimates or clock rate estimates. This is probably because the divergence time distribution, rather than tree balance *per se*, impacts these estimates.

## 5. Impact of mutation distribution on phylodynamic inference

**Figure 5.**
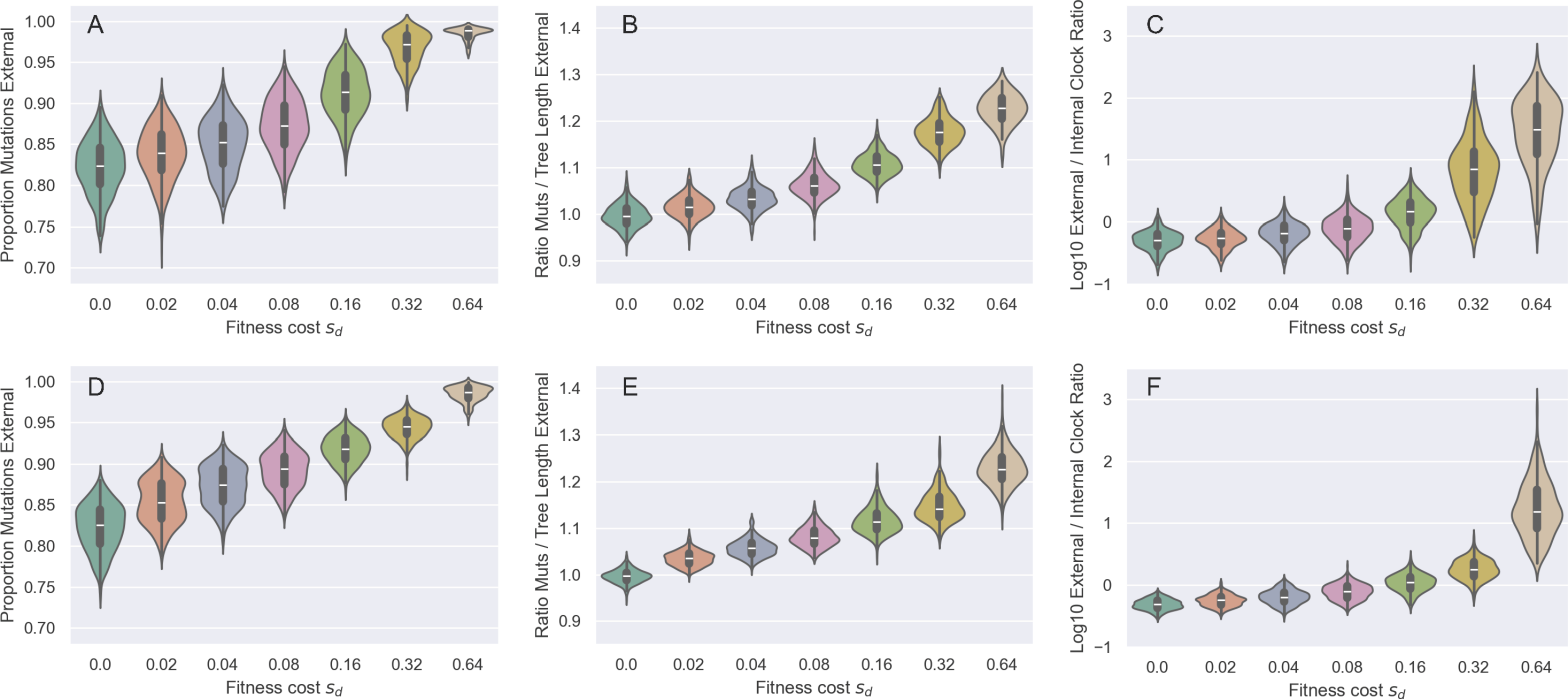
The impact of purifying selection on the distribution of mutations across a range of assumed deleterious fitness costs ($s_{d}$). (*a,d*) Proportion of mutations occurring along external branches. (*b,e*) The ratio of the proportion of external mutations to the proportion of external tree length. (*c,f*)The ratio of the true external clock rate versus the true internal clock rate. We note that at low values of $s_{d}$ the (log) ratio is slightly negative. This is because in our simulations mutations only occur at transmission events and we sample individuals upon recovery. The true clock rate is therefore slightly depressed along sampled external lineages. In (*a*)–(*c*), the mutation rate is m=0.30 per genome per transmission event. In (*d*)–(*f*), it is m=3.0 per genome per transmission event. Violin plots show distribution of summary statistics for 100 successful simulations of the forward model.

We next consider whether the distribution of mutations across viral phylogenies could bias phylodynamic inference in the case of incomplete purifying selection. To do this, we used the same simulations from §4, this time examining the distribution of mutations over the true genealogies. At the mutation rate reflecting empirical values for SARS-CoV-2 (m=0.3 mutations per genome per transmission), incomplete purifying selection had a very strong impact on the distribution of mutations (figure 5*a*–c). The proportion of mutations falling on external branches (i.e. singletons, $\eta_{e}/S$ in [31]) increases markedly with the strength of purifying selection (figure 5*a*). To see how selection shifts the distribution of mutations independent of distortions in branch lengths, we also compared the proportion of external mutations to the proportion of the tree that consisted of external branches. This ratio grows rapidly with the strength of purifying selection, demonstrating that proportionally more deleterious mutations occur on external branches even after accounting for changing branch lengths (figure 5*b*). The shift in the distribution of mutations across the tree also has a major impact on branch-specific clock rates. At the strongest levels of purifying selection ($s_{d}=0.64$) clock rates along external branches are one to two orders of magnitude higher than along internal branches (figure 5*c*).

We repeated these analyses for the simulations parametrized with an order of magnitude higher mutation rate (m=3.0; figure 5*d*–*f*). Again, we found that the proportion of mutations that lie on external branches increases with the fitness cost of deleterious mutations (figure 5*d*). The ratio of external mutations to external tree length again increases with increasing costs of deleterious mutations (figure 5*e*). Finally, clock rates along external branches are again higher than those on internal branches, although, at a given $s_{d}$, this ratio is lower at the higher mutation rate than at the lower mutation rate (figure 5*f* versus figure 5*c*). This is probably because purifying selection can more efficiently remove lineages with an excess of deleterious mutations when mutation rates are high.

**Figure 6.**
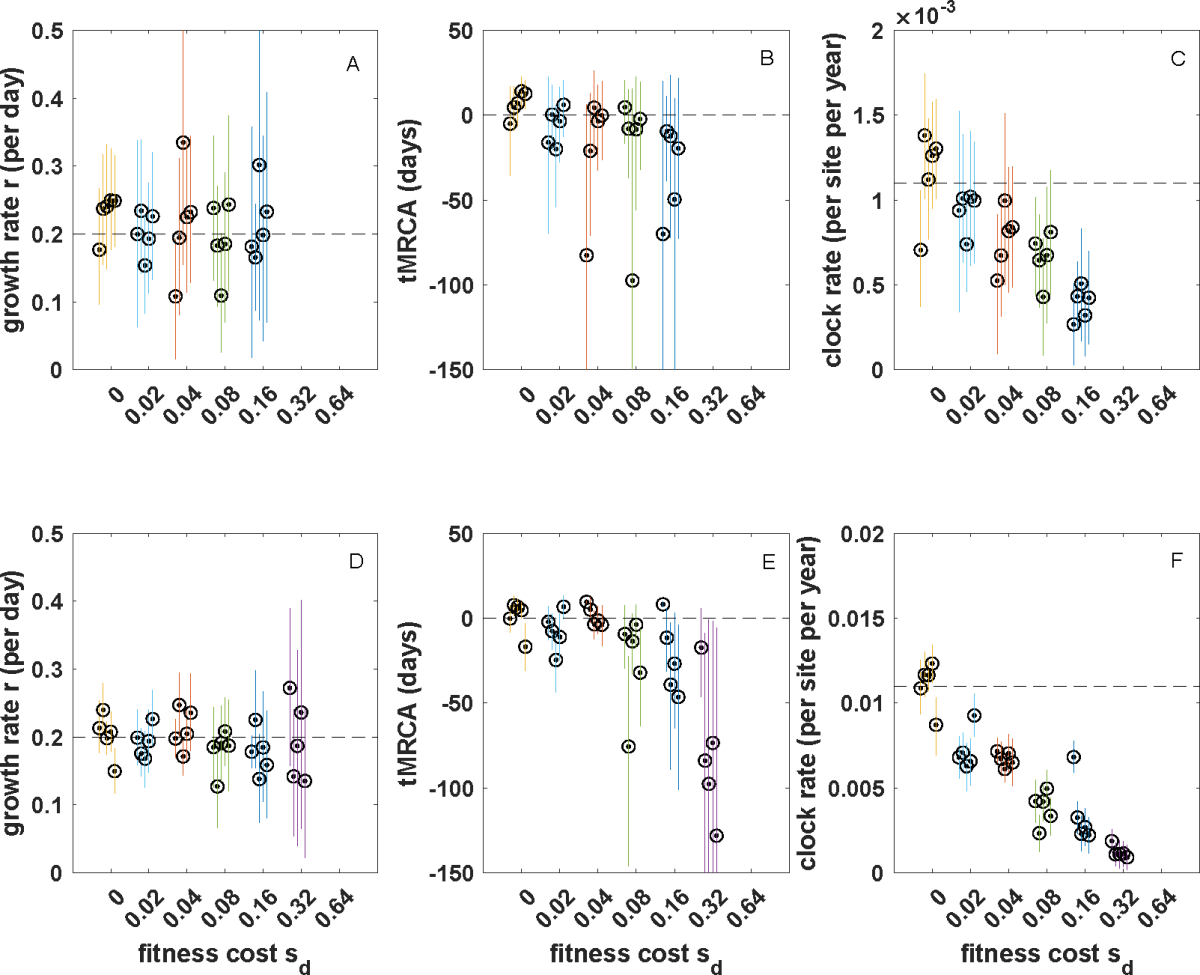
The impact of altered mutation distribution (jointly with tree shape) on phylodynamic inference across a range of assumed deleterious fitness costs. (*a), (d*) Estimated exponential growth rates. (*b), (e*) Estimated times of the most recent common ancestor. (*c),(f*) Estimated clock rates. In (*a*)–(*c*), the mutation rate m=0.3. In (*d)–(f*), the mutation rate m=3.0. At both mutation rates, inferences from five replicate simulations are shown for each fitness cost. As in figure 4, circles show mean estimates and lines show 95% HPD intervals. Dashed lines in panels (*a*,*c*,*d*, *f*) show true values. In panels (*b*) and (*e*), dashed lines show simulation start times of T=0 days. True tMRCA times may fall slightly later than T=0 days. With the exception of m=3.0 and $s_{d}=0.32$, MCMC chain length for all runs was $10^{9}$, with all effective sample sizes (ESS) exceeding 200 in all runs. Inference on sequences from m=3.0 and $s_{d}=0.32$ simulations combined 2–3 log files, each with an MCMC chain length of $10^{9}$ such that ESS exceeded 200 in all five replicates. Inference was not possible under a parametrization of m=0.3 and $s_{d}=0.32$, under a parametrization of m=0.3 and $s_{d}=0.64$ or under a parametrization of m=3.0 and $s_{d}=0.64$ owing to insufficient amounts of genetic variation between sampled individuals. Uninformative priors were used for all parameters. For ease of comparison across results, panels (*a*) and (*d*) have the same *y*-axis range and panels (*b*) and (*e*) have the same *y*-axis range.

Given this observed impact of incomplete purifying selection on the distribution of mutations on our simulated viral phylogenies, we next asked whether phylodynamic inference would still be able to accurately recover epidemiological parameter values. To address this question, we used the same set of true genealogies from §4, this time simulating sequences for each observed tip based on the true mutations that occurred along each branch. These sequences thus reflect the skewed distribution of mutations on the true genealogies as well as any changes in tree shape that incomplete purifying selection may have left in these genealogies. We then again applied the coalescent exponential model to these simulated sequences to determine whether there would be biases in growth rate or tMRCA estimates. Much to our surprise, we were again able to accurately infer exponential growth rates under both of these mutation rates (figure 6*a*,*d*). At the low mutation rate, we were also able to recover the time of the most recent common ancestor across the range of mutational fitness costs considered (figure 6*b*). The ability to accurately recover the tMRCA despite incomplete purifying selection is likely due to heterochronous sampling, with the earliest sequenced sample providing an upper bound for the tMRCA estimate. At the high mutation rate, starting at $s_{d}=0.08$, tMRCA estimates were biased towards the more distant past (figure 6*e*). At both mutation rates, inferred clock rates were lower the higher the fitness cost (figure 6*c*,*f*). These latter findings are unsurprising, given that purifying selection decreases the number of observed substitutions along a tree, particularly on internal branches. The biases in tMRCA estimates (figure 6*e*) may be a result of these lower clock rates: the tMRCA is most likely estimated to occur in the more distant past because we underestimate the clock rate, requiring more time to have elapsed between the tMRCA and the tips to explain the observed genetic distances between sequences.

## 6. Sensitivity to sampling times

The impact of incomplete purifying selection on viral phylogenies and phylodynamic inference may be dependent on when sampling occurs. For example, we may have only been able to accurately estimate epidemic growth rates in the face of strong purifying selection because we sampled viral lineages continuously through time, including early on when few deleterious mutations are segregating. Likewise, purifying selection may have the greatest impact on estimated clock rates when samples are only collected over a short period of time in the recent past [21,23,25,27]. We therefore explored how sensitive our results are to different sampling schemes in our m=0.3 mutation rate simulations. We conducted this sensitivity analysis by varying the time period over which samples were collected. As a point of comparison with our previous simulations where we sampled continuously over the full T=55 days of exponential growth, we considered cases where the sampling period was constrained to the final 20 days or the final 5 days of the simulation.

**Figure 7.**
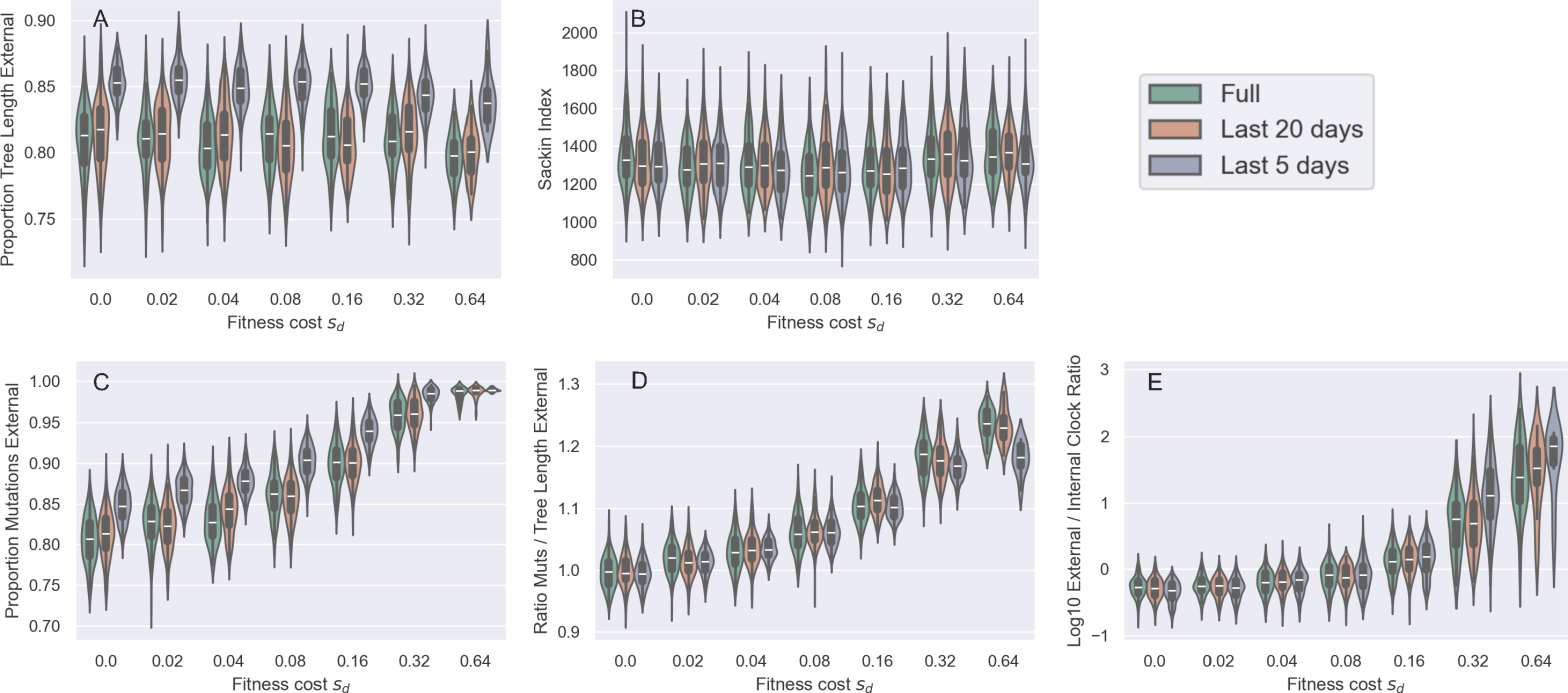
The impact of purifying selection on tree shape and the distribution of mutations when sampling occurs over different time periods. Violin plots are coloured according to whether samples were collected over the full epidemic (green), the final 20 days (orange) or the final 5 days (blue). (*a*) Proportion of tree length composed of external branches. (*b*) Sackin index of imbalance. (*c*) Proportion of mutations occurring along external branches. (*d*) The ratio of the proportion of external mutations to the proportion of external tree length. (*e*) The ratio of the true external clock rate versus the true internal clock rate. The mutation rate was m=0.30 per genome per transmission in all simulations. In all simulations, we sampled 200 individuals at random from the set of individuals that recovered over the sampling period. Because of this and the exponential growth of the infected population, there are always a larger number of individuals sampled towards the end of the time period considered.

As before, incomplete purifying selection has little impact on tree shape in terms of the proportion of external tree length or the Sackin index, regardless of the sampling period (figure 7*a*–*b*). External lineages become longer when sampling is restricted to the more recent past, but this is expected for any exponentially growing population where the population size is larger in the more recent past, resulting in more recently sampled lineages coalescing at a slower rate than lineages sampled at earlier time points. In contrast to tree shape, sampling in the more recent past tends to exacerbate the impact of purifying selection on the distribution of mutations. The proportion of singleton mutations falling on external branches increases the later sampling occurs, regardless of $s_{d}$ value (figure 7*c*), although at very high $s_{d}$ values nearly all mutations occur along external lineages regardless of when sampling occurs. As a result of this, sampling in the more recent past has a larger impact on the proportion of external tree length than on the proportion of external mutations when the fitness costs of mutations are very large (figure 7*a* versus figure 7c). Thus the ratio of the proportion of external mutations to the proportion of external tree length can actually decrease as sampling occurs later at large $s_{d}$ values (figure 7*d*). Nevertheless, clock rates remain higher along external lineages relative to internal lineages when sampling occurs in the more recent past (figure 7*e*), consistent with earlier empirical observations [25].

**Figure 8.**
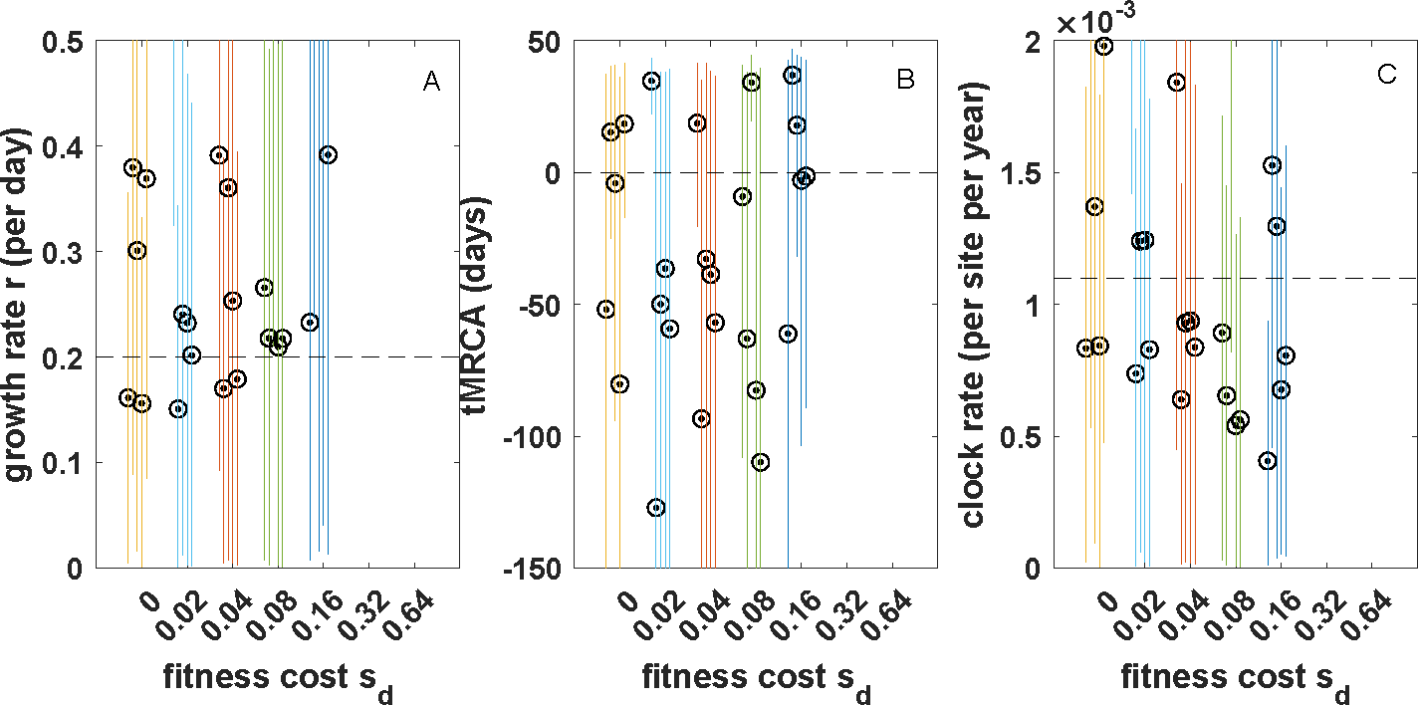
The impact of incomplete purifying selection on phylodynamic inference across a range of deleterious fitness costs, for simulations with a mutation rate of m=0.3 and sampling only individuals who recovered over last five days of the simulation. (*a*) Estimated growth rate. (*b*) Estimated time of the most recent common ancestor. (*c*) Estimated clock rate. All MCMC chain lengths were $10^{9}$ or combined across multiple log files to obtain ESS values exceeding 200. Uninformative priors were used for all estimated parameters.

To determine whether phylodynamic inference could still recover the true epidemiological parameter values when sampling of individuals was constrained to a short period of time, we again generated sets of sequences from 200 individuals sampled over the final five days of the simulations. As in figure 6, the sequences we generated reflected the mutation distributions of the true genealogies. We once more attempted to estimate the growth rate, tMRCA and clock rate under the coalescent exponential model. Again, to our surprise, we were able to accurately recover the growth rate r, the tMRCA and the true clock rate (figure 8). Although our 95% highest posterior density (HPD) intervals generally contained the true values of these parameters, these credible intervals were (as expected) much wider when individuals were sampled over a five-day period compared with when individuals were sampled over the full time period of the epidemic (figure 8 versus figure 6*a*–c). Owing to these wide credible intervals, it is also difficult to determine whether sampling over a highly constrained period of time indeed biases phylodynamic inference.

## 7. Discussion

Phylodynamic inference methods, including both coalescent-based and birth–death approaches, generally assume that all observed and ancestral viral genetic variation is fitness neutral. Here, we first briefly reviewed studies showing that this assumption is violated for the majority of studied viral pathogens, including those circulating in humans. These studies have generally found that incomplete purifying selection occurs in viral populations, resulting in transient circulation of sublethal deleterious mutations. We then reviewed existing findings from the population genetic literature that have assessed the impact of incomplete purifying selection on tree shape and mutation distribution. Based on these results, we anticipated that tree shape might be, under certain parametrizations, distorted from its neutral expectation in viral populations undergoing epidemic expansion. We further anticipated that the distribution of mutations across viral genealogies would be starkly impacted by incomplete purifying selection, with a shift in mutations towards the external branches of trees and away from internal branches. As a result of these expectations, we hypothesized that existing phylodynamic inference methods that assume neutral evolution could yield biased parameter estimates when applied to viral sequences sampled from populations that are subject to incomplete purifying selection. Because of the heavy use of phylodynamic inference during pandemic and/or lineage emergence, we evaluated this hypothesis in the context of an exponentially growing viral population.

We found that, under a parametrization that reflected values estimated for SARS-CoV-2 during its early 2020 viral expansion, tree shape was not substantially impacted by incomplete purifying selection across a broad range of considered mutational fitness costs (figure 3*a*–*b*). Phylodynamic inference applied to simulated sequence data generated by exclusively considering impacts on tree shape was also able to successfully recover the true epidemiological parameters (figure 4*a*–*c*). We then found that the distribution of mutations across trees indeed shifted substantially as mutations exacted an increasing fitness cost (figure 5*a*–*c*). Surprisingly, however, phylodynamic inference was able to nevertheless recover the true growth rate and the true tMRCA. Clock rates, as expected, were lower at higher mutational fitness costs, owing to purifying selection. At an order-of-magnitude higher mutation rate as well as at a reduced sampling period of five days, phylodynamic inference was still able to recover the true epidemiological parameters of interest, again, much to our surprise (figure 6*d*,*e*; figure 8*a*,*b*).

Our analyses involved a limited number of scenarios and many assumptions. First, we largely considered a single parametrization with a growth rate of r=0.2 per day and a mutation rate of m=0.3 mutations per genome per transmission event, although we did explore a considerably higher mutation rate scenario (m=3.0 mutations per genome per transmission event). We also considered only an epidemic invasion scenario, where a total of 200 individuals were sampled within 55 days of the start of the epidemic, although we also explored sensitivity to sampling over a more constrained period of time. As such, we are not certain of the generalizability of our results. It could be that under a different parametrization or under a different sampling scheme, incomplete purifying selection could bias phylodynamic estimates. For example, biases may be apparent when estimating the growth rate of an exponentially declining viral population subject to incomplete purifying selection. A more comprehensive analysis of how incomplete purifying selection impacts the shape of viral phylogenies and their mutation distribution is a topic of future work. Ideally, analytical solutions could be derived that would help us understand which epidemiological and mutation parameters affect when tree shape in particular is most impacted by incomplete purifying selection. Finally, we restricted ourselves to inference using the coalescent exponential model to examine the impact of incomplete purifying selection on phylodynamic inference. We anticipate that inference based on the birth–death model would yield similar results, but we have not performed these analyses. In sum, our analyses indicate that phylodynamic inference is not appreciably biased by the circulation of deleterious mutations that fall between ‘viable’ and ‘non-viable’. That epidemiological parameters estimated under neutral phylodynamic models align as closely as they do with their true values in our simulations despite pervasive incomplete purifying selection is, as Yule would put it ‘in fact better than one has any right to expect’.

## 8. Methods

### (a) Forward simulation model

To understand the impact of purifying selection on viral phylogenies, we simulated the epidemiological and evolutionary dynamics of an exponentially growing viral population experiencing deleterious mutations. For all simulations, we considered a spillover scenario with a single initially infected individual. We used parameters consistent with those for SARS-CoV-2 [47]. In the context of an epidemic, births and deaths of individuals represent transmission and recovery events, respectively. We set the birth rate of infected individuals to λ=0.3 per day and the death rate of infected individuals to δ=0.1 per day. These birth and death rates together determine the intrinsic growth rate of the infected population (r=λ−δ=0.2 per day), its doubling time (D=ln(2)/r≈3.5 days), as well as the basic reproduction number ($R_{0}=\lambda /\delta =3$).

We let mutations occur at birth, with the number of mutations being Poisson-distributed with mean m. We set the per genome, per transmission event mutation rate to m=0.3, again consistent with empirical estimates for SARS-CoV-2 [48]. With a SARS-CoV-2 genome size of 29 903 nucleotide sites and a birth rate of λ=0.3 per day, this mutation rate yields a substitution rate of approximately $1.1\times 10^{-3}$ substitutions per site per year under a neutral model of evolution:


(8.1) $$0.3\frac{mutations}{genome\cdot transmission}\times \frac{1}{29903}\frac{genome}{sites}\times 0.3\frac{transmissions}{day}\times 365.25\frac{days}{year}\approx 0.0011\frac{substitutions}{site\cdot year}.$$

All mutations within a given simulation had the same fixed fitness cost ($s_{d}$). We varied fitness effects across simulations and considered $s_{d}$ values of 0, 0.02, 0.04, 0.08, 0.16, 0.32 and 0.64, with the $s_{d}=0$ simulations serving as the neutral control. We assumed multiplicative fitness effects of mutations, such that a virus with k mutations had a fitness of $w=(1-s_{d}{)}^{k}$. We did not consider epistasis of any kind.

We forward simulated for 55 days (from T=0 days to T=55 days) and randomly sampled 200 individuals upon recovery from each simulation. However, because of demographic stochasticity, the cumulative number of infections reached by T=55 days differed between simulations. We therefore conditioned our simulations on surviving to T=55 days and reaching a sufficient size such that 200 individuals could be sampled without replacement over the chosen sampling interval. For each unique model parametrization, simulations were repeatedly performed until 100 successful realizations meeting these conditions were obtained.

### (b) Model implementation for tree shape and mutation distribution analyses

We implemented our forward model in SLiM v. 4.01 [49] to obtain simulated viral phylogenies reflecting the true ancestry of all sampled individuals as well as the mutations that fall on the branches of these phylogenies. While SLiM simulates under a discrete time model with a fixed time step of Δt, we approximate the continuous time dynamics of our forward model by sampling the number and type of events occurring over each time step according to the propensities given by their instantaneous rates under the analogous continuous time model. Specifically, we draw the number of transmission events occurring over a discrete time step from a Poisson distribution with mean equal to λIΔt (where *I* is the current number of infected individuals) and draw the number of recovery events occurring over this time step from a Poisson distribution with mean equal to δIΔt. The identity of the parent/source of each new infection was randomly sampled independently with probability proportional to the parent’s fitness w, i.e. by multinomial sampling with replacement. We chose a small discrete time step Δt=0.01 days to ensure minimal deviation of the simulated discrete time process from the analogous continuous time process.

SLiM was used to record the full genealogical relationships among all individuals in the infected population. Subsequent to each simulation, tskit v. 0.4.1 [50] was used to process these trees to only reflect the ancestry of sampled individuals. Two tree shape statistics were calculated from these extracted subtrees: the proportion of the total tree length that falls on external branches and the Sackin index of imbalance. The Sackin index of imbalance is defined as the sum of a tree’s leaves’ depths [51]:


(8.2) $$\begin{array}{ccc} & I_{s}^{n}=\sum_{i=1}^{n}N_{i}, & \end{array}$$

where $N_{i}$ is the number of internal nodes in the path from leaf i to the root and n is the total number of leaves in the tree.

Beyond ancestry information, SLiM also recorded information on the number and timing of mutations that occurred at transmission events. Information on the occurrence of these mutations was used, together with the extracted subtrees, to quantify the distribution of mutations on the sampled genealogies.

Python code for running and processing the SLiM simulations is available at [52].

### (c) Model implementation for phylodynamic inference

For phylodynamic inference, an exact birth–death model, implemented in Matlab v. R2023A, was instead used to simulate exponential viral growth. Ancestry, along with occurrences of mutations, were similarly tracked in these forward simulations and the true genealogical relationships between sampled individuals were extracted from the full genealogical relationships that the model simulation stored.

From the genealogical trees extracted for the set of sampled individuals, we generated simulated sequences and saved these as fasta files that could be loaded into BEAST1 [46]. We simulated sequences in two different ways. First, in order to see how purifying selection impacts phylodynamic inference through its effect on tree shape but independent of its impact on the distribution of mutations, we scattered mutations on the extracted genealogies, with the number of mutations on a branch being a Poisson random variable with mean given by the branch length (in units of time) times the generation interval (1/λ) times the per genome per transmission event mutation rate m. This is equivalent to setting the mean number of substitutions on the branch to the product of the substitution rate ($1.1\times 10^{-3}$ substitutions per site per year), the number of sites (29 903) and the branch length (in units of years). Second, in order to see how purifying selection impacts phylodynamic inference through its effect on both tree shape and mutation distribution, we instead simply used information from the forward simulations to quantify the number of mutations that occurred along each of the branches in the extracted genealogy.

In both cases, once the mutations were placed on the extracted genealogy, we generated sequences for each of the 200 sampled tips in the following manner. We first set the root node to the SARS-CoV-2 Wuhan-Hu-1 reference isolate (GenBank accession number NC_045512) and then assumed an HKY (Hasegawa–Kishono–Yano) model of sequence evolution with a transition : transversion ratio of 3 and nucleotide frequencies set to their empirical frequencies. In simulating sequences up to the sampled tips, we assumed that a mutation, once encountered on a branch, occurred at a random site in the SARS-CoV-2 genome and resulted in a mutation that was sampled probabilistically based on the transition/transversion rates in a HKY model of sequence evolution. Sequences at the tips were saved to a fasta file, with the header including the time at which the sampled individual recovered. Matlab code for the exact birth/death model simulation, for extraction of the genealogical relationships among sampled individuals and for simulating viral sequences is also available at [52].

We applied the coalescent exponential model [45] implemented in BEAST v. 1.10.4 [46] to the simulated sequence data from the 200 sampled individuals. We specified tip dates, assumed a strict molecular clock, an HKY model of sequence evolution with a transition : transversion ratio of 3 (κ=6) and empirical nucleotide frequencies. We also used uninformative priors.

## Data Availability

Computer code for reproducing the results presented in this paper is available at [52].
